# Diagnostic profile of Pediatric T-Lymphoblastic Leukemia and its correlation with post-induction disease status

**DOI:** 10.12669/pjms.42.(ICON26).15686

**Published:** 2026-04

**Authors:** Zeenat Amna Azhar, Neelum Mansoor, Saba Jamal

**Affiliations:** 1 Dr. Zeenat Amna Azhar, MBBS, Resident Hematology, Department of Hematology, Indus Hospital and Health Network, Karachi, Pakistan; 2 Dr. Neelum Mansoor, MBBS, FCPS. Consultant Hematologist, Department of Hematology, Indus Hospital and Health Network, Karachi, Pakistan; 3 Dr. Saba Jamal, MBBS, Diplomate American Board of Hematology, Consultant Hematologist, Director Clinical Laboratories & Blood Transfusion Services. Department of Hematology, Indus Hospital and Health Network, Karachi, Pakistan

**Keywords:** Acute lymphoblastic leukemia, Countries, Flowcytometry, Immunophenotype, MRD, Low- and Middle-Income

## Abstract

**Objective:**

To determine the diagnostic profile of T-ALL in pediatric patients and correlate the association of the clinical characteristics with early disease outcome determined through minimal/measurable residual disease (MRD) status, tested after the induction phase of chemotherapy at day-35.

**Methodology:**

This retrospective cohort study was conducted at the Hematology Department of Indus Hospital and Health Network, Karachi, (IHHN) from October 2023 to December 2024 including 75 newly diagnosed T-ALL patients aged 1-16 years, who presented to our facility and were under treatment in Pediatric Oncology services.

**Results:**

Median age of patients was 9.52 years, with male to female ratio of 4:1. Most frequent presenting complaint was fever 92%. Mediastinal mass was present at 34.7% and CNS involvement in 4%. Immunophenotypic profile showed aberrant CD13 and CD79a expression in 1.3%. Following early treatment, 81.3% were steroid good responders; 1.3% expired before day 8, 2.7% expired before day=35. After induction of chemotherapy, 92% achieved morphological remission whereas 74.6% were MRD negative.

**Conclusion:**

In LMICs like Pakistan, pediatric T-ALL patients usually presents with advanced disease. However, risk stratification employing clinical features and early treatment outcome leading to intensified multi-agent chemotherapy administration ultimately achieved disease remission in majority of the patients, similar in comparison to local and international data.

## INTRODUCTION

T-cell acute lymphoblastic leukemia (T-ALL) is an aggressive hematologic malignancy originating from immature T-cell progenitors, constituting approximately 10–15% of childhood acute lymphoblastic leukemia (ALL) cases.^1^,^2^ T-ALL, although less frequent than B-cell ALL, exhibits a unique clinical and biochemical profile, frequently linked to elevated leukocyte counts at diagnosis, substantial mediastinal masses, central nervous system involvement, and a rapid disease progression.^3^ Recent advancements in multi-agent chemotherapy have enhanced outcomes in childhood T-ALL, achieving survival rates of 80% in affluent settings.^4^ Nevertheless, the outcomes in low- and middle-income countries (LMICs), including Pakistan, are still inadequate because of challenges such as delayed diagnosis, restricted access to diagnostic and prognostic tools, and treatment abandonment. These factors contribute to elevated rates of relapse and induction failure.

In Pakistan, T-ALL constitutes between 12–15% of newly diagnosed pediatric ALL patients in tertiary care facilities.^5^,^6^ Most patients present late with advanced disease, characterized by a large tumor burden, organomegaly, and comorbidities such as tumor lysis syndrome or superior mediastinal syndrome. Absence of systematic risk stratification via immunophenotypic and molecular profiling, coupled with restricted access to minimal residual disease (MRD) testing, impedes personalized treatment.^7^ This leads to suboptimal outcomes, particularly in children exhibiting high-risk characteristics who may benefit from early treatment intensification or allogeneic stem cell transplantation.

Immunophenotyping is essential for confirming the diagnosis of T-ALL and distinguishing it from other lymphoid or myeloid neoplasms. Furthermore, post-induction MRD evaluation serves as a vital prognostic indicator, closely linked to likelihood of relapse and long-term survival outcomes.^8^ Recognizing clinical and diagnostic characteristics at presentation and examining their association with MRD status can facilitate early risk stratification and therapeutic decision-making, thereby enhancing treatment outcomes in pediatric T-ALL.

There is a dearth of local information on clinical and diagnostic spectrum, and post-induction outcomes of pediatric T-ALL in LMICs. The objective of this study is to present the clinical and diagnostic characteristics of children diagnosed with T-ALL at a tertiary care pediatric oncology center in Pakistan. It also aimed to investigate correlation between these features and post-induction MRD status to identify potential indicators for early risk prediction and outcome assessment. The results may be used to inform policy-level decisions, risk-adapted therapy, and diagnostic prioritization for pediatric leukemia management in resource-limited settings.

## METHODOLOGY

This retrospective cohort study was conducted at the Hematology Department of IHHN, Karachi, from October 2023 to December 2024.

### Ethical approval:

The Institutional Review Board (IRB) provided ethical approval (IRB # IHHN_IRB_2024_06_008; dated January 21, 2025), and patient consent was waived as study is retrospective in nature, deemed low-risk, and required no additional sampling.

All patient data were handled with strict confidentiality. Identifiable information was removed before analysis, and data were coded to ensure anonymity. Access to the dataset was restricted to study investigators only. The study included 75 pediatric patients aged one to 16 years who were all newly diagnosed with T-ALL and received treatment at our facility during study period. The individuals under one year or over 16 years of age, as well as those diagnosed with B-Lymphoblastic Leukemia (B-ALL), Acute Myeloid Leukemia (AML), or Lymphoma, were excluded. Clinical and diagnostic data were obtained retrospectively from the hospital’s Health Management Information System (HMIS). Extracted data encompassed patient demographics, clinical characteristics, and laboratory parameters.

A complete blood count (CBC) was performed with the Sysmex XN 1000 automated hematology analyzer, followed by a manual peripheral smear examination to confirm presence of blast cells. Immunophenotyping was performed by eight-color multiparameter flowcytometry on peripheral blood or bone marrow aspirate specimens. The antibody panel used includes TdT, CD34, CD99, CD1a, cytoplasmic CD3, surface CD3, CD5, CD7, CD4, CD8, CD2, CD10, CD79a, CD13, CD33, CD73, and CD123. Marker expression was classified as positive or negative by comparing it with internal control populations, in accordance with College of American Pathologists (CAP) recommendations. Patients were assessed for Tumor Lysis Syndrome (TLS) using Cairo- Bishop Criteria. Central Nervous System (CNS) involvement was ascertained by a baseline Cerebrospinal Fluid (CSF) differential (D/R) following lumbar puncture; a positive CNS status indicated presence of blasts in the CSF, while a negative status implied that no blasts were seen. Mediastinal mass (symptomatic/asymptomatic) was identified on chest x-ray. MRD testing was performed by eight-color flowcytometry on BD FACS Canto II analyzer using FACS Diva software by acquiring 500,000 events. The in-house limit of detection (LOD) for the MRD assay is 0.01% which is validated as per the College of American Pathologists (CAP) protocol using the appropriate panel of antibodies. The quality control (QC) of the analysis system was based on instrumental daily QC and internal positive and negative controls. Analysis was performed on the strategy of Leukemia Associated Immunophenotype (LAIP) detection. Interpretation was made according to CAP guidelines. Immunophenotypic shifts that may occur between diagnosis and follow-up were carefully evaluated during MRD analysis. The diagnostic immunophenotype for each case was documented in detail, including both aberrant and lineage-associated markers. During MRD evaluations, potential antigen modulation or loss (e.g., CD10, CD34, CD7, CD99, or TdT) was considered. Gating strategies were adjusted to account for these changes, and interpretation relied on the identification of leukemia-associated immunophenotypes (LAIPs) in combination with “different-from-normal” (DfN) approaches. When uncertainty arose, results were reviewed by two independent hematopathologists to ensure accuracy.

**Fig.1:**
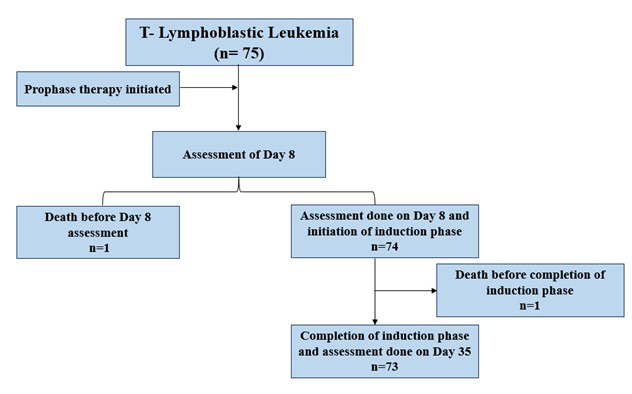
Schematic representation of the study cohort, showing the number of T-ALL patients enrolled at diagnosis and subsequent patient numbers at key study timepoints.

All diagnostic and MRD testing were performed in the IHHN flowcytometry laboratory on same instrument using the same panel of antibodies. All patients underwent prophase therapy with prednisolone for one week, followed by a response assessment on Day eight using CBC and peripheral smear analysis. Fig.1 shows schematic representation of our study cohort. Patients (n=74) were classified as good or poor responders using an absolute blast count of <1x10e9/L or >1x10e9/L, respectively in the Day-8 CBC in the day-8 CBC. However, one patient expired before completion of prophase. High-risk induction chemotherapy was administered in accordance with the ALL protocol established by the Pakistan Society of Pediatric Oncology (PSPO 2020). Post-induction response was evaluated using bone marrow morphological remission and MRD testing via flowcytometry on Day 35 in 73 patients (excluding two deaths before completion of induction chemotherapy). MRD was deemed negative if less than 0.01% of blasts were identified in bone marrow aspirate.

### Statistical analysis:

Data were analyzed using IBM SPSS Statistics version 23.0. Categorical variables (e.g., gender, clinical features, MRD status, steroid response) were presented as frequencies and percentages. Continuous variables e.g. age, Total Leukocyte Count (TLC), Hemoglobin (Hb) level etc. were summarized using means and standard deviations or medians with interquartile ranges, as appropriate.

To assess the relationship between baseline clinical or diagnostic variables and MRD status, different statistical tests were applied including Chi-square test or Fisher’s exact test for categorical variables and independent samples t-test or Mann–Whitney U test for continuous variables depending on data distribution. A p-value of <0.05 was considered statistically significant. Due to a small proportion of patients with positive MRD i.e. 17 (from a statistical standpoint), there was limited power for multivariate analysis; therefore, only univariate association was assessed to avoid model over-fitting.

## RESULTS

A total of 75 pediatric patients, newly diagnosed with T-ALL, were included. Various demographic and clinical features are shown in Table-I. The average age of the patients was 9.52 ± 3.96 years. Gender distribution showed a clear male dominance, with 78.7% of participants being male and 21.3% female. The most common presenting complaint was fever n=69 (92%). Chest x-ray revealed presence of mediastinal mass in n=26 (34.7%) of the patients. Note that out of 26 patients having a mediastinal mass, dyspnea was observed in only n=13 (17.3%) patients. Among the presenting blood counts, median TLC was 95x10^9^/L. The TLC was less than 50x10^9^/L in n=26 (34.7%), 50-100x10^9^/L in n=13 (17.3%), and more than 100x10^9^/L in n=36 (48%) of the cases. Median blast percentage was 82% (61-91), implying the aggressive nature of the disease. CNS status was determined in all patients and was reported positive i.e. presence of leukemic cells in CSF in n=3 (4%) of the patients. Tumor Lysis Syndrome was observed in n=9 (12%) cases.

**Table I:** Demographic and Clinical characteristics of patients (n=75)

Variables	St Statistics
***Demographic Feature***	
Age, Years Median (IQR)	9.0 (7.0-13.0)
≤10 Years, n (%)	43 (57.3)
>10 Years, n (%)	32 (42.7)
Gender (M/F), n (%)	59 (78.7)/16 (21.3)
***Clinical Presentation***	
Fever, n (%)	69 (92.0)
Generalized Weakness, n (%)	20 (26.7)
Weight Loss, n (%)	13 (17.3)
Bleeding or Bruising, n (%)	29 (38.7)
Dyspnea, n (%)	13 (17.3)
Lymphadenopathy, n (%)	67 (89.3)
Bone pain, n (%)	34 (45.3)
Visceromegaly, n (%)	60 (80.0)
CNS Positive, n (%)	3 (4.0)
Pleural effusion, n (%)	7 (9.3)
Mediastinal Mass, n (%)	26 (34.7)
Tumor Lysis Syndrome, n (%)	9 (12.0)
***Complete Blood Count***	
Hemoglobin	6.9 (5.0-8.5)
<7 g/dl, n (%)	39 (52.0)
>7 g/dl, n (%)	36 (48.0)
Total Leukocyte Count (x10^9^/L)	95.0 (35.0-371.0)
<50, n (%)	26 (34.7)
50-100, n (%)	13 (17.3)
>100, n (%)	36 (48.0)
Platelets (x10^9^/L)	26.0 (15.0-52.0)
<100, n (%)	62 (82.7)
>100, n (%)	13 (17.3)
Blast%, n (%)	82.0 (61.0-91.0)
***Diagnostic Specimen***	
Peripheral Blood, n (%)	62 (82.7)
Bone Marrow Aspirate, n (%)	13 (17.3)

IQR: Interquartile ratio, CNS: Central nervous system.

Out of 75 cases, flowcytometry was performed on peripheral blood in n=62 (82.7%) cases while in n=13 (17.3%) it was done on bone marrow aspirates as described in Table-I. The immunophenotypic characterization of the leukemic population is shown in Table-II. Immaturity of the blasts was established using a panel of markers showing positivity of TdT (90.7%), CD99 (81.3%), CD1a (58.7%) and CD34 (33.3%). Whereas T-lymphoid lineage of the blasts was defined by positive expression of cytoplasmic CD3 (100%) and other T-lineage markers. An aberrant expression of CD79a and CD13 was noted in (1.3%) cases.

**Table II:** Immunophenotypic profile of T-lymphoblastic leukemia (n=75).

Flow Cytometry Markers	Positive, n (%)	Negative, n (%)
TdT	68 (90.7)	7 (9.3)
CD34	25 (33.3)	50 (66.7)
CD99	61 (81.3)	14 (18.7)
CD1a	44 (58.7)	31 (41.3)
Cytoplasmic CD3	75 (100.0)	0
Surface CD3	72 (96.0)	3 (4.0)
CD4	52 (69.3)	23 (30.7)
CD8	49 (65.3)	26 (34.7)
CD5	68 (90.7)	7 (9.3)
CD7	73 (97.3)	2 (2.7)
CD2	75 (100.0)	0
CD79a	1 (1.3)	74 (98.7)
CD10	0	75 (100)
CD13	1 (1.3)	74 (98.7)
CD33	0	75 (100.0)
CD73	1 (1.3)	74 (98.7)
CD123	6 (8.0)	69 (92.0)

TdT: Terminal deoxynucleotidyl transferase, CD: Cluster of differentiation.

**Fig-2:**
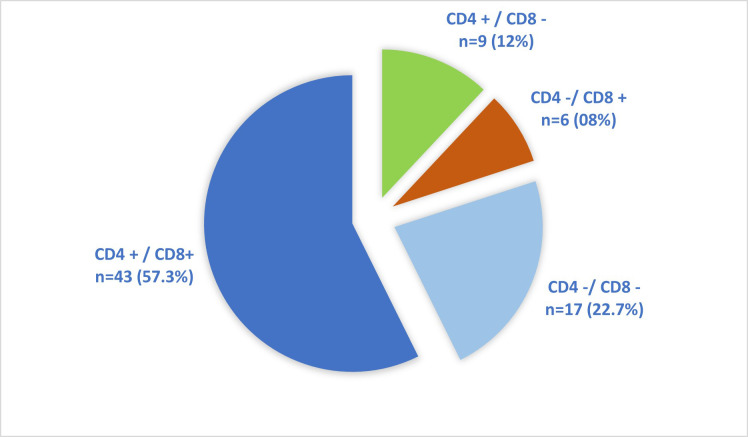
Distribution of CD4 and CD8 expression among T-ALL patients (n=75).

**Fig-3:**
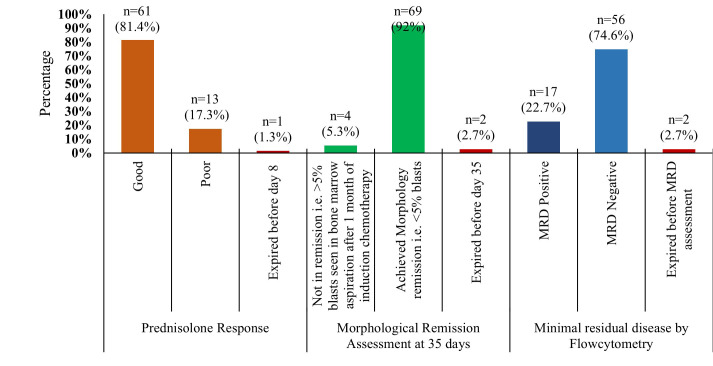
Assessment of treatment response and disease remission Early treatment response was assessed on Day-8 in 74 patients as one patient had expired while the status of the disease remission in the terms of morphology and minimal residual disease was assessed on Day 35 in 73 patients as one more patient had expired before reaching this milestone

The spectrum of immunophenotypic expression of CD4 and CD8 among study participants is depicted in Fig.2. The key clinical outcomes related to the response to prednisolone, morphological remission status at 35 days, and presence of MRD in patients following induction chemotherapy are shown in Fig.3. A significant percentage of patients exhibited a favorable response to prednisolone n=61 (81.4%) and attained complete morphological remission (CR) by the end of induction therapy n=69 (92%), indicating an overall optimal response to initial treatment. MRD was assessed by flowcytometry, providing a more sensitive measure of treatment response. Among the cohort, n=56 (74.6%) was MRD-negative, suggesting a deep remission with no detectable leukemic cells.

On comparison of baseline clinical and laboratory features with post-induction MRD status (Table-III), no significant differences were observed in age, gender distribution, hemoglobin levels, white blood cell count, or blast percentage at presentation. Morphological remission was not achieved in 23.5% of MRD-positive patients, whereas all MRD-negative patients attained remission (p=0.001, hence establishing significant association between the two variables). Notably, 13 (76.5%) of MRD-positive cases had achieved morphological remission, highlighting that a substantial proportion of patients with apparent remission by morphology still harbored measurable residual disease. Other variables, including the presence of mediastinal mass, tumor lysis syndrome, and prednisolone response, did not show a statistically significant association with post-induction MRD status.

**Table III:** Comparison of Some Baseline Clinical and Laboratory Features with Post-induction MRD status (n=73)

Variables		Residual Disease Present n=17	No Residual Disease n=56	P-Value
Age (Years)		9.0 (6.5-13.0)	10.0 (7.0-13.0)	0.596^δ^
Gender	Male, n (%)	13 (76.5)	44 (78.6)	1.000^£^
	Female, n (%)	4 (23.5)	12 (21.4)	
Total Leukocyte Count		55.2 (18.1-170.0)	102.4 (36.6-403.3)	0.114^δ^
Blast		80.0 (28.0-88.5)	82.5 (61.3-91.0)	0.435^δ^
Mediastinal Mass	Present, n (%)	14 (82.4)	35 (62.5)	0.152^β^
	Absent, n (%)	3 (17.6)	21 (37.5)	
Morphological remission	Achieved, n (%)	4 (23.5)	0	0.001^δ*^
	Not achieved, n (%)	13 (76.5)	56 (100.0)	
Prednisone response	Good, n (%)	12 (70.6)	49 (87.5)	0.135^£^
	Poor, n (%)	5 (29.4)	7 (12.5)	

δ Mann-Whitney U test;

£ Fisher-Exact test;

β Pearson’s Chi-Square test;

* statistically significant.

## DISCUSSION

We explored the diagnostic profile and early disease outcome in pediatric T-ALL patients, which is a higher-risk and less frequently studied entity compared to B-ALL. Additionally, in Pakistan and other LMICs, late presentations, restricted laboratory capacity, and sporadic MRD testing can alter both the clinical spectrum and treatment outcomes, underscoring the need for locally generated data. Establishing associations between baseline features and early outcomes in local cohorts could facilitate early risk-adapted therapeutic decisions, particularly in settings where MRD testing is not available.

In our cohort, we observed male predominance with a median age of 9.5 years at diagnosis, consistent with previously reported epidemiological data from Pakistan.^9^,^10^ Clinically, most patients presented with high-risk features, including severe anemia, elevated TLC, and mediastinal mass leading to respiratory distress or pleural effusion, while central nervous system involvement was identified in only a minority of cases.

All cases in our cohort were diagnosed by flow cytometry (FCM) using a broad panel of lineage-specific and non-lineage markers to identify aberrant co-expression of myeloid or B-lymphoid antigens on T-cells, early T-cell precursor ALL (ETP-ALL), mixed phenotype acute leukemias, and leukemia-associated immunophenotypes (LAIPs). The use of an extended diagnostic panel also enhanced the sensitivity and specificity of MRD detection, particularly in accounting for therapy-induced phenotypic shifts.^11^ In our study, aberrant expression of CD13 and CD79a was observed in 1.33% of cases. By comparison, Jamal et al. reported aberrant CD79a expression in 11.1% and CD13 in 8.1% of 209 T-ALL cases, a difference most likely attributable to sample size.^10^ Regarding CD4/CD8 expression patterns, 57.3% of our cases demonstrated co-expression of CD4 and CD8, 22.7% were double-negative, 12% were CD4-positive/CD8-negative, and 8% were CD8-positive/CD4-negative. In contrast, Gupta et al. documented CD4/CD8 co-expression in 39.3% of cases, double negativity in 32.8%, CD4-positive/CD8-negative in 21.3%, and CD8-positive/CD4-negative in 6.6% of T-ALL cases.^12^

During the early treatment course, Day eight response to corticosteroid therapy serves as an easily accessible marker of treatment sensitivity. In our study, 81.33% patients were good responders, 17.34% poor responders, and 1.33% expired due to intraventricular hemorrhage before Day eight assessment. Arshed et al. studied the clinical profile and early treatment outcome of 121 children with T-ALL and found that 77.7% of the patients were good responders, and 19.8% were poor responders.^9^ Likewise, Wei *et al* studied 74 pediatric T-ALL patients, which revealed steroid good response in 84.6% of patients and poor response in 15.6% of the cases.^13^ The results of these studies agree with our study. A poor steroid response has been associated with an unfavorable treatment outcome, expecting a higher minimal residual disease (MRD) burden subsequently.^14^ However, the predictive value of steroid response is not absolute; a subset of good steroid responders may still harbor MRD positivity. This indicates that Day eight blast counts should not be used alone, but as part of a multimodal risk assessment algorithm.

Post-induction MRD assessment has been incorporated into most international treatment protocols as a cornerstone of early risk stratification, as it identifies high-risk patients who may appear to be in morphological remission.^15^ Our study revealed that 13 patients who achieved morphological CR at Day 35 were MRD-positive by FCM, underscoring the limitations of morphology alone in detecting residual disease. This discrepancy likely reflects limited sensitivity of light microscopy and its high interobserver variability, as well as the inability of morphological assessment to detect low-level residual leukemic populations.^16^ MRD monitoring, by contrast, provides reproducible and quantifiable data that correlates more reliably with relapse risk, thereby enabling timely therapeutic escalation or enrollment in clinical trials for poor responders.^17^,^18^ In resource-limited settings where MRD assessment is unavailable, clinicians often rely on baseline clinical and laboratory features such as high presenting TLC, blast percentage, CNS involvement, or immunophenotypic subtype for the initial risk stratification. However, these parameters do not reliably reflect residual disease burden. In our cohort, no significant association was observed between demographic, clinical, biological, or immunophenotypic features and post-induction MRD status (p > 0.05), a finding that is consistent with the report by Meraj et al., who conducted a similar study in pediatric B-ALL patients within the same setting.^19^

Introduction of intensified chemotherapy protocols designed for high-risk ALL has significantly improved remission rates in pediatric T-ALL. At the end of induction, 69 out of 73 evaluated patients (94.5%) achieved morphological complete remission (CR), while 13 of these patients remained MRD-positive by flow cytometry. Thus, 74.6% of patients achieved both morphological remission and MRD negativity. However, cohorts from low- and middle-income countries (LMICs) frequently report inferior remission and event-free survival (EFS) rates, when compared with large studies from high-income countries (HICs), where five year survival rate now exceeds 90%.^20^ These disparities are most likely attributable to delayed referrals, malnutrition, limited supportive and intensive care facilities, and treatment abandonment, all of which contribute to higher treatment-related mortality.^21^

### Limitations

Limitations of our study were that only early disease outcome were studied and follow-up of diagnosed patients till the completion of therapy was not done as it takes two to three years. Additionally, ETP-ALL cases (diagnosed as per WHO classification of Hematolymphoid Tumors, 2022 criteria) could not be included as this entity is rare and no case was detected during our study duration. Unlike B-ALL, conventional cytogenetic and FISH testing have limited utility in pediatric T-ALL, as there are no well-defined cytogenetic subtypes with clinical or therapeutic significance. Therefore, these tests are not routinely recommended. Additionally, performing such analyses does not provide a cost–benefit advantage for treatment planning or protocol selection. Although certain high-risk or poor-prognostic genomic alterations like NOTCH1 and PTEN have been described in the literature, molecular testing for these mutations (via Next Generation Sequencing) and for T-cell receptor gene rearrangements is not currently available in our laboratory.

## CONCLUSION

Our study provides one of the few detailed characterizations of pediatric T-ALL in Pakistan, highlighting its predominance in older boys, frequent presentation with high-risk clinical features, and diagnostic value of extended flowcytometry panels. Early treatment responses, including steroid sensitivity and post-induction MRD, were comparable to international reports. Importantly, no baseline demographic or immunophenotypic features reliably predicted MRD status, reinforcing the central role of MRD testing in risk stratification. These findings underscore a need for improved access to MRD monitoring, long-term follow-up, and genomic profiling in local cohorts to guide risk-adapted therapy and optimize survival.

### Recommendations

Further prospective multicenter collaborative studies with long-term follow-up will be helpful to confirm prognostic value of clinical and diagnostic features for relapse and survival outcomes. Additionally, genomic profiling of local T-ALL cases could uncover novel biological markers of risk, paving the way for targeted therapies in our patients.

### Authors Contribution:

**ZAA:** Study concept, study design, literature search, data collection, analysis, and interpretation.

**NM: S**tudy design, questionnaire design, data interpretation, and provided feedback through critical manuscript review.

**SJ:** Literature search and manuscript writing.

All authors have read the final version and are responsible and accountable for the accuracy and integrity of the work.
